# An evaluation of selected chemical, biochemical, and biological parameters of soil enriched with vermicompost

**DOI:** 10.1007/s11356-020-10981-z

**Published:** 2020-10-13

**Authors:** Sebastian Wojciech Przemieniecki, Anita Zapałowska, Andrzej Skwiercz, Marta Damszel, Arkadiusz Telesiński, Zbigniew Sierota, Anna Gorczyca

**Affiliations:** 1 Faculty of Environmental Management and Agriculture, Department of Entomology, Phytopathology and Molecular Diagnostics, University of Warmia and Mazury in Olsztyn, Prawocheńskiego 17, 10-720 Olsztyn, Poland; 2 College of Natural Sciences, Institute of Agricultural Sciences, Land Management and Environmental Protection, University of Rzeszów, Cwiklinskiej 1a, 35-601 Rzeszów, Poland; 3 Department of Pests Management, Research Institute of Horticulture in Skierniewice, Pomologiczna 18, 96-100 Skierniewice, Poland; 4 Faculty of Environmental Management and Agriculture, Department of Bioengineering, West Pomeranian University of Technology in Szczecin, Słowackiego 17, 71-434 Szczecin, Poland; 5 Faculty of Environmental Management and Agriculture, Department of Forestry and Forest Ecology, University of Warmia and Mazury in Olsztyn, Pl. Łódzki 2, 10-727 Olsztyn, Poland; 6 Faculty of Agriculture and Economics, Department of Microbiology and Biomonitoring, University of Agriculture in Krakow, Mickiewicza 21, 31-120 Krakow, Poland

**Keywords:** Vermicompost, Larch, Soil, Chemical properties, Microbiota, Enzymes

## Abstract

The aim of this study was to assess the changes in chemical and microbial properties and enzymatic activity of soil enriched with vermicompost derived from household waste. The vermicompost was tested in the rhizosphere of *Larix* decidua seedlings cultivated in 10-L pots in: (i) nursery soil (as the control), (ii) soil with 10% v/v vermicompost, and (iii) with 20% v/v vermicompost. The impact of vermicompost was assessed in terms of soil C/N ratio; bacterial, fungal, and nematode counts; and enzymatic activity. It was found that vermicompost increased the C/N ratio from 21 to 32, as well as the content of nitrate from 78 to 134 mg kg^−1^, of ammonium from 14 to 139 mg kg^−1^, of phosphorus from 92 to 521 mg kg^−1^, and of potassium from 142 to 1912 mg kg^−1^, compared with the control soil. The abundance of beneficial bacteria was increased (from 8.61 × 10^7^ to 37.9 × 10^7^), along with decreases in microbiological ratios of fungi and bacteria (e.g. fungi/Bacillus from 0.18818 to 0.00425). A significant 2- to 4-fold increase was observed compared with the control in the number of beneficial nematodes belonging to bacterivorous, fungivorous, and predatory groups with no change in the abundance of plant-parasitic nematodes. Addition of vermicompost brought about a change in soil enzyme activity. Vermicompost reduced the activity of alkaline phosphatase only. Both doses of vermicompost led to an increase in the activity of acid phosphatase, inorganic pyrophosphatase, dehydrogenases, β-glucosidase, and urease. Only the higher dose had an effect on increasing the activity of o-diphenol oxidase and proteases. No significant change was observed for nitrate reductase. Also, the presence of antibiotics produced by bacteria was detected depending on the dose of vermicompost, e.g. iturin (*ituC*) and bacillomycin (*bmyB*) were found in soil with a dose of 20% v/v vermicompost. Overall, vermicompost produced from household waste can be an excellent organic fertilizer for larch forest nurseries.

**Electronic supplementary material:**

The online version of this article (10.1007/s11356-020-10981-z) contains supplementary material, which is available to authorized users.

## Introduction

Vermicomposting is a sustainable technology for utilizing organic waste as a result of which natural fertilizer applied to soil improves its yielding properties. The production and application of vermicompost directly counteract the threats inherent to the modern bioeconomy, i.e. the generation of a huge amount of organic waste and the loss of soil yielding properties (FAO and ITPS 2015; Silpa et al. 2018). Rapid biodegradation of organic waste during vermicomposting is a result of the interaction between earthworms and microorganisms.

Earthworms cause fragmentation and conditioning of the substrate. Epigeic earthworms (sensu Bouché 1977) are the most suitable for producing vermicompost as they live in organic horizons, feed primarily on decaying organic matter, and are the most efficient in biodegrading organic waste and releasing nutrients into the soil (Lim et al. 2016). Among the epigeic earthworms, *Eisenia fetida* is the most commonly used in vermicomposting because of its worldwide distribution, and it is resilient and has a wide temperature tolerance (Edwards 2004). In addition to rapidly fragmenting and processing the biodegradable fraction (6–18 h), earthworms cause the secretion of enzymes, eliminate harmful microorganisms, and contribute to the development of beneficial microorganisms (actinides, fungi) in the soil (Flegel and Schrader 2000; Pathma and Sakthivel 2012). Microorganisms are responsible for the stabilization of biological processes in soil solution, and soil enzymes produced by microorganisms participate in the circulation of organic and inorganic matter. In a state of equilibrium of the environment (homeostasis), in addition to naturally occurring plant defence mechanisms, soil microorganisms play a key role in supporting these mechanisms, providing nutrients, and producing growth stimulants.

Vermicompost produced by earthworms can improve soil physical properties such as aggregate stability, total porosity, air and water permeability related to decreasing in bulk density, and penetration resistance. Also, soil water-holding capacity can be increased with the addition of vermicompost. Within chemical properties, it was found that nitrate-nitrogen, phosphorus, calcium, magnesium, zinc, copper, and iron content, as well as electrical conductivity, increased. In comparison with mineral fertilizers, vermicompost produces significantly greater increases in soil organic carbon and some plant nutrients (Bachman and Metzger 2007; Weber et al. 2007; Aksakal et al. 2016; Lim et al. 2016). One gramme of vermicompost contains up to 2000 billion bacteria belonging to the Bacterioides, Gammaproteobacteria, Deltaproteobacteria, Actinobacteria, Alphaproteobacteria, Betaproteobacteria, Chloroflexi, Firmicutes, Acidobacteria, Gemmatimonadetes, Verrucomicrobia, and Planctomycetes (Pathma and Sakthivel 2012; Neher et al. 2013). During vermicomposting, the nutrients locked up in the organic waste are changed into simple and more readily available forms, such as nitrate or ammonium nitrogen; exchangeable phosphorus; and soluble potassium, calcium, and magnesium in the worm gut (Atiyeh et al. 2002). Microorganisms, as the second producer of vermicompost after earthworms, provide therefore soil environment which has very favourable properties for the development of plants and determining their healthiness (Lehman et al. 2015; Jacoby et al. 2017). There is some data available on the effect of vermicomposting in improving plant resistance to pests (Arancon et al. 2005; Cardoza 2011; Hussain et al. 2017), and in particular to soil nematodes, an area in which conventional methods of control are really weak (Szczech et al. 1993; Blouin et al. 2005; Rajiv et al. 2013). The bacteria that are responsible for the production of effective antibiotics against phytopathogens include the three most widely described groups: Pseudomonadaceae, Actinomycetes, and *Bacillus* spp. They can be immobilized in soil mineral matter, be inactivated or degraded by microorganisms, and are sensitive to unfavourable environmental conditions (pH, temperature, water availability) (Burns et al. 2013; Schimel et al. 2017).

Vermicompost can be used in all farming systems, especially in organic farming, where there are problems with fertilizing crops. As a consequence, it is also suitable for use in households too (Venkatesh and Eevera 2008; Sinha et al. 2011; Lim et al. 2015). Unlike agricultural and horticultural crops, forest crops and especially forest nurseries have not yet been well studied in terms of the benefits of using vermicompost. There are a few results which indicate the positive effect of vermicompost on germination and early growth of seedlings (Lazcano et al. 2010), as well as on morphological and physiological quality of four genotypes of pine (Atik 2014; Atik and Yilmaz 2014). In turn, Pérez-Piqueres et al. (2018) observed the lack of a clearly beneficial effect of vermicompost as a root-growth promoter of four genotypes, also of pine. To date, the properties of forest nursery soils enriched with vermicompost have not been assessed, also with regard to other tree species.

The aim of the study was to assess the microbial diversity and enzymatic activity in soil amended with household vermicompost—to recognize a pool of soil organisms in the substrates and the number of nematodes against the background of the basic physicochemical properties of soil and soil enzymes. The results obtained were used as a basis for determining the usefulness of vermicomposts in larch forestry nursery production and the implementation of IPM principles.

## Materials and methods

### Soil and vermicompost

The experiment was carried out near the Baltic coast (54° 35′ 29″ N, 18° 27′ 42″ E). Soils used in this study are classified as Eutrophic Cambisols, with loam texture (Kabała et al. 2019). A quantity of 300 L of soil was obtained from a forest nursery. The humidity of the samples before the test was 60% of the maximum water volume.

Vermicompost was produced by the earthworm (*E. fetida*) processing fresh household waste (waste from lawnmower clippings, fruit waste—mainly apples and pears, vegetable waste—mainly potatoes and carrots) in a continuous, long-term decomposition process (6 months) in a concrete composter. Vermicompost was obtained from the upper part, free from earthworms, and was mixed with soil to create 10% v/v (variant V10) and 20% v/v (variant V20) mixtures. Soil without vermicompost was used as the control. Ten-litre pots were filled with the prepared substrates in 3 replicates for seedlings of larch (*Larix decidua* Mill.). Larch seedlings came from the Domatowo Forest Nursery (Wejherowo Forest District, Pomeranian Voivodeship, Poland) and were of 30 cm high; measurements were performed in the rhizosphere of seedlings after 12 months of cultivation.

### Chemical properties of samples

The pH of the samples and the salinity were measured according to Kabała and Karczewska (2019): pH in H_2_O and KCl by the potentiometric method; salinity by electrical conductivity; phosphorus and potassium by the Egner-Riehm method; magnesium by the Schachtschabel method; calcium and chlorine by atomic absorption spectrophotometry method; total nitrogen by the Kiejdahl method; total organic carbon spectrophotometrically after oxidation; ammonium (N–NH_4_) by Nessler’s colorimetric method; nitrate (N–NO_3_) by the colorimetric method with phenol-2,4 disulphonic acid.

### Isolation of genetic material and qPCR execution

The assessment concerned the basic groups of microorganisms that are indicators of the condition of the soil environment. Samples were ground in a mortar and screened through a 2-mm sieve. One hundred milligrammes of material was transferred to a 2-ml tube containing glass beads and lysis buffer, and then homogenized in a TissueLiser LT (Qiagen, Germany). Cell lysis was carried out for 5 min at maximum speed. DNA from the samples were isolated from soil and mixtures of soil with vermicompost (V10 and V20) using the Soil DNA Purification Kit (EURx, Poland). The quality of the isolated genetic material was ~ 1.8 (absorbance index 260/280). Isolation for each substrate was performed in two series.

To assess the number of bacteria, the real-time TaqMan method was used with the BAC338F and BAC805R primers, and the BAC516F probe (Yu et al. 2005) using a Maxima Probe qPCR Master Mix 2× (Thermo Fisher Scientific, USA). The Sybr Green technique with the NSI1 and 58A2R primers was used for fungi determination (Martin and Rygiewicz 2005). Maxima SYBR Green qPCR Master Mix 2× (Thermo Fisher Scientific, USA) was used to prepare the reaction. Determination of the number of toxin-producing fungi of the *Fusarium* genus was performed with the SYBR Green methods with the Tox5-1 and Tox5-2 primer sets, described by Schnerr et al. (2001). The determination of the toxicity number of *Penicillium* spp. and *Aspergillus* spp. was made using primers and probes developed by Suanthie et al. (2009). Enumeration of the 16S rDNA copy gene of *Clostridium* was performed according to Song et al. (2004) using a Probe-I GTGCCAGCAGCCGCGGTAATACG (*Clostridium* cluster I) forward primer, CI-F1, and reverse primer, CI-R2. The number of the *phlD* gene of PGPB (plant growth-promoting bacteria) *Pseudomonadaceae* was determined according to the Hu et al. (2016) primer set. To determine *Bacillus* spp., 16SBACF and 16SBACR primers were used (Mora et al. 2011). Both Pseudomonadaceae and *Bacillus* were amplified with the Maxima Sybr Green qPCR Master Mix 2× (Thermo Fisher Scientific, USA).

DNA from pure cultures of *Bacillus subtilis*, *Pseudomonas putida*, *Clostridium perfringens*, *Fusarium culmorum*, *Penicillium chrysogenum*, and *Aspergillus niger* were used as standards for their respective domains. The PCR efficiency of the reactions was successively between 0.90 and 1.02 (*R*^2^ between 0.98 and 1).

The number of actinomycetes was determined based on the dilution method using Actinomycete Isolation Agar (Sigma-Aldrich, USA).

### Nematode abundance

The nematode population density was analyzed by a method described by Zapałowska and Skwiercz (2018) for four trophic groups of nematodes: plant-parasitic, bacterivorous, fungivorous, and predatory nematodes. From each of the samples (substrate), a subsample of 100 cm^3^ was taken and blended. Nematodes from the soil samples were collected by centrifugation. After centrifugation, nematodes were killed by 6% hot formalin. After processing to glycerin by the Seinhorst (1962) method, permanent slides were made. Plant-parasitic nematodes were identified for genus according to the Brzeski key (1998), whereas bacterivorous, fungivorous, and predatory nematodes were identified according to the Andrássy key (2007).

### Detection of genes responsible for the production of antibiotics effective against phytopathogens

Antibiotics demonstrating antifungal activity were measured together with the primers used and the size of the expected PCR product. Genes coding antimicrobial non-hermospheric polypeptide synthetase/type I polyketide synthase PPKS-I produced by Actinomycetes were detected for using primers and methods described by Ayuso-Sacido and Genilloud (2005). PCR for the *srfAA*, *bacA*, *fenD*, *bmyB*, and *ituC* genes was performed according to Mora et al. (2011) for the *phlD* and *hcnAB* genes; detection was performed according to Svercel et al. (2007) and McSpadden Gardener et al. (2001), respectively. All reaction products were detected by gel electrophoresis (UVP GelDoc-it, UVP, LLC, Canada).

### Determination of soil enzyme activities

The activity of enzymes involved in P-, C-, and N-cycles was measured using a UV-1800 (Shimadzu, Japan) spectrophotometer. The analysis of P-cycle enzymes included determination of the activity of acid and alkaline phosphatases (ACP and ALP) and inorganic pyrophosphatase (IPP). The activity of ACP (EC 3.1.3.2) and ALP (EC 3.1.3.1) was assayed with the Tabatabai and Bremner (1969) method. The enzymatic activity was determined colorimetrically at a wavelength of 400 nm. IPP (EC 3.6.1.1) activity was determined according to the procedure proposed by Dick and Tabatabai (1978). Orthophosphate released by IPP activity was extracted with sulphuric acid and determined photometrically at 700 nm after colorization with ammonium molybdate.

The analysis of C-cycle enzymes included determination of the activity of dehydrogenases (DHA), *o*-diphenol oxidase (oDPO), and β-glucosidase (GLU). The activity of DHA (EC 1.1.1) was determined by the method of Casida et al. (1964). The oDPO (EC 1.10.3.1) activity was determined according to the procedure presented by Perucci et al. (2000). The activity of GLU (EC 3.2.1.21) was determined with the method developed by Eivazi and Tabatabai (1988).

The analysis of N-cycle enzymes included determination of the activity of nitrate reductase (NR), proteases (PROT), and urease (URE). NR (EC 1.6.6.1) activity was determined according to Abdelmagid and Tabatabai (1987), using KNO_3_ as a substrate. The activity of PROT (EC 3.4.4) was determined using casein as a substrate (Ladd and Butler 1972) along with sodium caseinate. URE activity (EC 3.5.1.5) was determined with the method developed by Kandeler and Gerber (1988) using urea as a substrate. Released ammonium was extracted with a potassium chloride solution and determined colorimetrically by a modified Berthelot reaction at 660 nm.

### Statistics

The Kruskal-Wallis test at *p* = 0.05 was used in the statistical calculations for microbiological tests. For statistical calculations for the biometric and enzymatic tests, the univariate ANOVA with the Levene homogeneity test of variance, Neuman Keuls, and Tukey HSD were used at *p* = 0.05 (Statistica 13.1, StatSoft, https://www.statsoft.pl/statistica_13). The correlation was calculated and visualized using SigmaPlot 13 (Systat Software, http://www.sigmaplot.co.uk/products/sigmaplot); principal component analysis was performed in XLSTAT (Addinsoft, https://www.xlstat.com).

## Results

The vermicompost caused soil alkalization—especially at the higher dose, with high K and P content (Table 1). Vermicompost increased the C/N ratio to 32 and 25 percentage in treatments V10 and V20, respectively. Also, the content of nitrate, ammonium, and magnesium was increased. Almost all favourable changes were dependent on the dose of vermicompost except for a lower nitrate content in V20. In the case of V20, strong nitrate assimilation could have occurred while ammonium remained in the soil sorption complex. Biological fixation and nitrification could have also determined nitrogen changes. Additionally, an enzymatic activity indicates that vermicompost additives did not affect the denitrification process (Table 5).

**Table 1:** Chemical properties of control and vermicompost-amended soil after 12-month *Larix decidua* seedling cultivation (V10—10% v/v vermicompost/soil and V20—20% v/v vermicompost/soil)

Parameter	Control soil	Vermicompost (input)	V10	V20
pH–H_2_O	6.82	7.18	7.07	7.74
pH–KCl	6.6	7.0	6.7	7.0
Electrical conductivity (mS cm^−1^)	2.08	2.42	2.4	2.66
Nitrate (mg kg^−1^)	77.7	200	134	90.6
Ammonium (mg kg^−1^)	13.8	68.8	95	139
Phosphorus (mg kg^−1^)	92.2	618	358	521
Potassium (mg kg^−1^)	142	1796	1199	1912
Calcium (mg kg^−1^)	2680	1411	2046	1728
Magnesium (mg kg^−1^)	144	370	306	375
Chlorine (mg kg^−1^)	326	190	376	348
Organic carbon (%)	3.38	5.09	2.03	2.21
Total nitrogen (%)	0.21	0.24	0.15	0.19
C/N ratio	16/1	21/1	32/1	25/1

Vermicompost increased the number of bacteria and fungi up to 86% of the variance. The number of toxin-forming *Penicillium* spp. increased significantly in V10, while in the V20, the population of these fungi was significantly reduced in comparison with control soil (Table 2). It was found that toxicogenic *Fusarium* spp. occurred sporadically and the addition of vermicompost reduced it further. The Pseudomonadaceaea family was stable and did not change significantly. *Bacillus* spp. strongly increased in the substrate with the higher addition of vermicompost (V20) and became predominant. The relatively large *Clostridium* spp. population in soil increased 7 to 9 times in both treatments. The lower dose of vermicompost was more favourable for the development of Actinomycetes than the higher dose. The ratio of total Fungi/Bacteria remained constant after the addition of vermicompost (Table 3). Depending on the dose of vermicompost (higher dose–greater change) the Fungi/Pseudomonadaceae and Fungi/Actinomycetes ratios changed. A higher dose of vermicompost reduced the Fungi/Pseudomonadaceae; Fungi/*Bacillus*; and *Penicillium* to Actinomycetes, *Bacillus*, *Clostridium*, and Pseudomonadaceae ratios.

**Table 2:** Abundance of selected groups of microorganisms colonising control and vermicompost-amended soil after 12-month *Larix decidua* seedling cultivation (copy gene·100 mg^−1^ of substrate; *CFU g^−1^). Different letters following the values represent significant differences between group HSD test, *p* < 0.05

Group	Control soil		V10		V20	
Total Bacteria	8.61 × 10^7^	b	14.2 × 10^7^	b	37.93 × 10^7^	a
Total Fungi	0.61 × 0^4^	b	1.56 × 10^4^	b	4.42 × 10^4^	a
*Penicillium* spp.	3.96 × 10^3^	b	5.31 × 10^3^	a	0.10 × 10^3^	c
*Fusarium* spp.	13	a	0	b	2	b
Pseudomonadaceae	2.03 × 10^6^	b	3.59 × 10^6^	a	3.01 × 10^6^	a
*Bacillus* spp.	0.003 × 10^6^	b	2.86 × 10^6^	b	10.4 × 10^6^	a
*Clostridium* spp.	1.05 × 10^5^	b	9.11 × 10^5^	a	7.38 × 10^5^	a
Actinomycetes*	4.67 × 10^6^	b	14.8 × 10^6^	a	8.53 × 10^6^	b

**Table 3:** Microbiological ratios of the soil as influenced with different concentration of the vermicompost after 12-month *Larix decidua* seedling cultivation

Ratio	Control soil	V10	V20
Fungi/Bacteria	0.00007	0.00011	0.00012
Fungi/Pseudomonadaceae	0.00302	0.00433	0.01470
Fungi/*Bacillus*	0.18818	0.00544	0.00425
Fungi/*Clostridium*	0.05802	0.01708	0.05991
Fungi/Actinomycetes	0.00131	0.00105	0.00518
*Penicillium*/Pseudomonadaceae	0.00195	0.00148	0.00003
*Penicillium/Bacillus*	0.12173	0.00186	0.00001
*Penicillium/Clostridium*	0.03753	0.00583	0.00014
*Penicillium*/Actinomycetes	0.00085	0.00036	0.00001

Only the number of plant-parasitic nematode individuals remained at the same level after the addition of vermicompost. A significant increase in the number of nematodes belonging to bacterivorous, fungivorous, and predatory groups was observed in the substrate with vermicompost compared with the control soil. In the present study, the most common type of nematodes were bacterivorous, and their dominance in the soil (about 49%) increased in vermicompost-soil mixtures to about 65% of the share of all observed nematodes in soil samples. The low presence of predators despite the increase in numbers did not change overall. Fungivorous nematodes decreased from 38% to 30% with the addition of vermicompost (Table 4).

**Table 4:** Main groups of nematode abundance (pcs·100 cm^−3^ of substrate) in control and vermicompost-amended soil after 12-month *Larix decidua* seedling cultivation. Different letters following the values represent significant differences between group HSD test, *p* < 0.05. (*PPN* plant-parasitic nematodes, *BN* bacterivorous nematodes, *FN* fungivorous nematodes, *PN* predatory nematodes)

Group	Soil		V10		V20	
PPN	60	a	50	a	48	a
BN	270	b	900	a	1180	a
FN	185	b	420	a	420	a
PN	14	b	30	a	52	a

While the V10 treatment increased ACP, DHA, GLU, and ERE activities in relation to the control soil, the V20 stimulated the activity of the majority of the soil enzymes determined involved in the transformation of phosphorus, carbon, and nitrogen compounds. Only in the case of NR were there no significant changes in activity. In contrast, ALP activity was significantly reduced after using higher dose of vermicompost. In addition, when comparing the effects of individual doses of vermicompost, significant differences were found between the activity of ALP, DHA, GLU, URE, and PROT. Significantly different results in comparison with the control soil were shown in V10 (without oDPO, PROT, and NR). However, in the V20 mixture only, NR was not significantly improved after vermicompost treatment (Table 5).

**Table 5:** Enzyme activity as influenced by different concentration of the vermicompost after 12-month *Larix decidua* seedling cultivation. Different letters following the values represent significant differences between group HSD test, *p* < 0.05. (*ALP* alkaline phosphatase (mg p-NP·kg^−1^ substrate·h^−1^), *ACP* acid phosphatase (mg p-NP·kg^−1^ substrate·h^−1^), *IPP* inorganic pyrophosphatase (mg P-(PO_4_)_3_·kg^−1^ substrate·h^−1^), *DHA* dehydrogenases (mg TPF·kg^−1^ substrate·h^−1^), *oDPO* o-diphenol oxidase (mmol oxidized catechol·kg^−1^ substrate·h^−1^), *GLU* β-glucosidase (mg p-NP·kg^−1^ substrate·h^−1^), *URE* urease (mg N–NH_4+_·kg^−1^ substrate·h^−1^), *PROT* proteases (mg tyrosine·kg^−1^ substrate·h^−1^), *NR* nitrate reductase (mg N–NO_2_·kg^−1^ substrate·h^−1^))

Enzymes group	Type	Control soil		V10		V20	
P-cycle	ALP	401.81	a	386.47	a	306.06	b
	ACP	348.18	b	440.56	a	453.48	a
	IPP	254.86	b	289.37	a	302.29	a
C-cycle	DHA	7.03	c	12.42	b	14.98	a
	oDPO	11.19	b	11.41	b	12.33	a
	GLU	180.90	c	193.67	b	207.44	a
N-cycle	URE	14.88	c	18.89	b	20.76	a
	PROT	2.94	b	3.08	b	3.33	a
	NR	192.73	a	192.87	a	195.12	a

It has been shown that the gene responsible for the production of the active 2,4-DAPG (2,4-diacetylphloroglucinol) antibiotic, encoded by the *phlD* gene, is present in each variant (strong band) of the experiment. Pseudomonadaceae and the *hcnAB* hydrocyanic acid coded by this gene show proven antimicrobial activity and have not been demonstrated in the control soil, but in V10, and especially in the V20 variant, its presence is already noticeable. Another PCR duplex showed the detection of two genes involved in the synthesis of most antibiotics by actinomycetes, regardless of the variant. For *Bacillus* spp., five antibiotics described by Mora et al. (2011) were detected, though in both the substrates with vermicompost only. For iturin (*ituC*), the gene was detected in V20 only; for bacillisin (bacA), it was present in V10 and V20; for fengycin (*fenD*), a positive result was found in the V10 and V20 variants; for surfactin (*srfAA*), it was present in V10 and V20; and for bacillomycin (*bmyB*), it was present in V20 only (A1, Supplementary material).

**Fig. 1:**
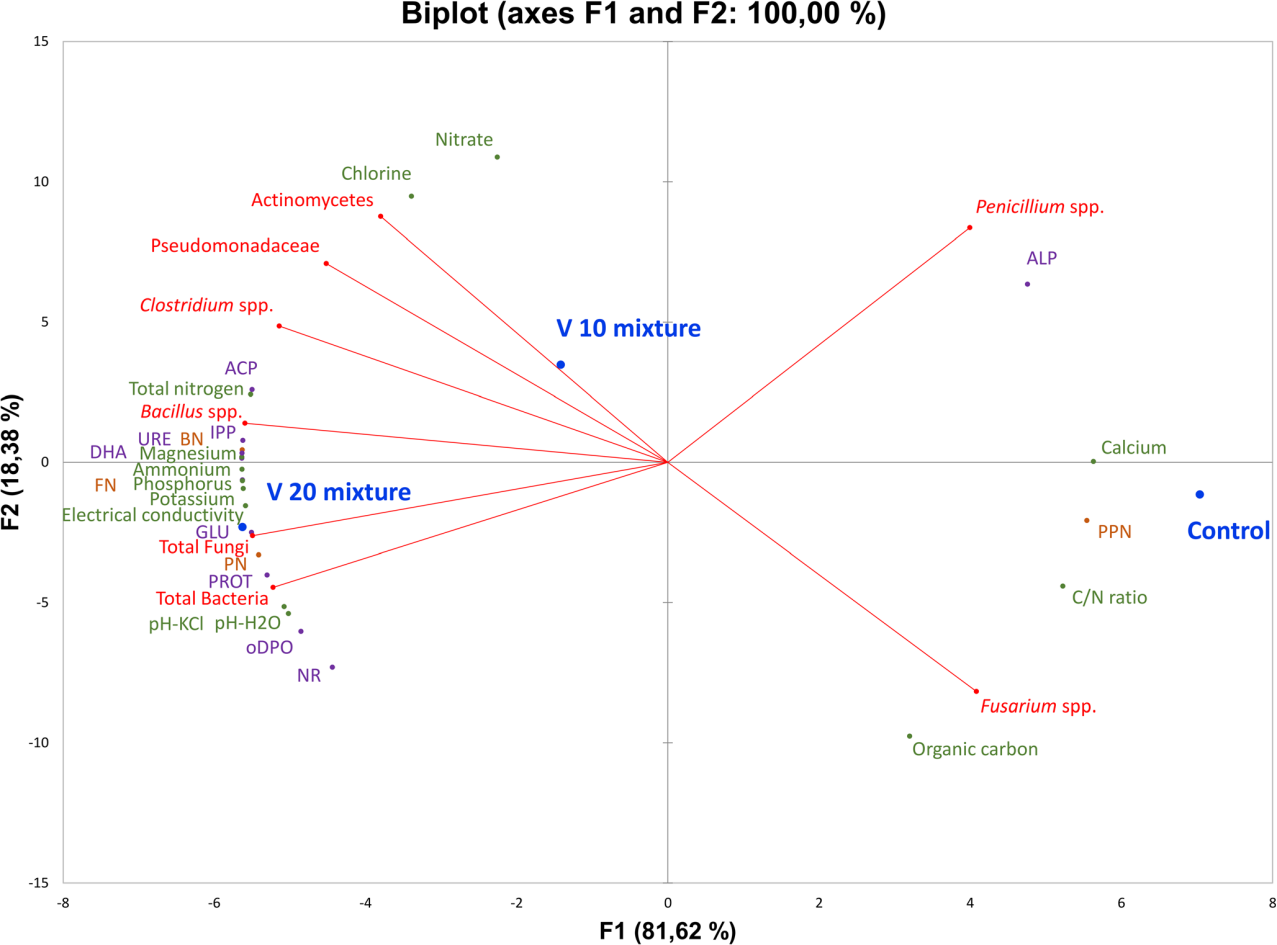
Biplot of the principal component analysis (PCA) for the variation of parameters for tested vermicopost, control soil, V10, and V20. Colours separate groups of parameters. EC electrical conductivity, PPN plant-parasitic nematodes, BN bacterivorous nematodes, FN fungivorous nematodes, PN predatory nematodes, ALP alkaline phosphatase, ACP acid phosphatase, IPP inorganic pyrophosphatase, DHA dehydrogenases, oDPO o-diphenol oxidase, GLU β-glucosidase, URE urease, PROT proteases, NR nitrate reductase

The hierarchical grouping analysis (Fig. 1) showed that each group was essentially different (indicated by the cutoff line), although the difference between the two substrates with the vermicompost and the control soil was more than twice as high as in the vermicompost-assisted substrates. Significant differences in the V10 and V20 were confirmed due to the lack of correlation between the parts of the variables (e.g., increased amount and the number of *Penicillium* spp. or Pseudomonadaceae in the community).

Results of the PCA analysis showed that the F1 component explains up to 86% of the variation in variance. Altogether, we compared 24 variables (4 for nematodes, 9 for enzymes, and 8 for microorganisms). The first dimension shows a qualitative separation between control soil and both vermicompost substrates. However, the V10 substrate has positive values for both component axes and V20 has negative values in relation to the F1 axis, and is positive for F2. For the control soil, the most significant correlations were found between the content of carbon, *Fusarium*, C/N ratio, PPN, and calcium, as well as between *Penicillium* spp. and ALP. For variant V10, there were correlations between nitrate, chlorine, Actinomycetes, Pseudomonadaceae, and *Clostridium* spp. and, to a large extent, with ACP and nitrogen. A constant relationship with the control soil and the less-assisted V10 substrate was observed for toxinogenic *Penicillium* and ALP. The group of correlated objects in the V10 substrate was Actinomycetes, Pseudomonadaceae, *Clostridium* spp., ACP, bacterivorous nematodes, *Bacillus* spp., fungivorous nematodes, IPP, URE, and DHA. The V20 variant was distinct from the others due to the creation of a large group of positively correlated variables consisting of GLU, total fungi, PR, PROT, total bacteria, pH (H_2_O and KCl), oODP, NR, salinity, potassium, phosphorus, fungivorous nematodes, nitrate, URE, magnesium, DHA, BF, IPP, *Bacillus* ssp., nitrogen, and ACP.

## Discussion

Vermicomposting in small-scale models like ours, at a low cost, can process organic household waste, which can be used to improve soil fertility (Pirsaheb et al. 2013; Pereira Mde et al. 2014). Presented research with *Larix* rhizosphere has confirmed favourable changes, especially in the case of the supply of potassium, phosphorus, and magnesium, as well as soil alkalization. Ten to 20% addition of vermicompost from household organic waste significantly improves the chemical properties of the soil and is an optimal alternative to chemical fertilization in forest nurseries. But the main observation from the research was that the addition of vermicompost improved the soil microbiome properties. The fungi-to-bacteria frequency ratio was not changed, although there was an increased level of some desired bacteria.

Not only household waste is a good substrate for vermicomposting, but also the improvement of microbial properties is found for vermicompost produced from different wastes. Vermicomposting of white grape marc resulted in a rich, stable bacterial community with functional properties that may aid plant growth (Kolbe et al. 2019). Yasir et al. (2009) reported that Proteobacteria, Bacteroidetes, Verrucomicrobia, Actinobacteria, and Firmicutes predominated in vermicompost from paper sludge. The activity of chitinolytic enzymes and enzymes degrading fungal cell walls was also detected, which was confirmed for the dangerous *Fusarium moniliforme* pathogen, whose population decreased after using vermicompost. In other studies, the analysis of the antifungal potential of vermicompost from *E. fetida* showed the dominance of *Bacillus* (57% in the bacterial population) and *Pseudomonas* (15%), the order Pseudomonadales (e.g., *Acinetobacter* 5%), and the group Actinomycetes (*Microbacterium* sp. 12%, *Arthrobacter*, and *Rhodococcus*), and several representatives of the Xandomonadales order. Many strains which are part of the microbial community of vermicompost belong to the PGPB (plant growth-promoting bacteria) group of bacteria with huge plant-supporting properties. These strains can produce protease, cellulase, lipase, xylanase, chitinase, amylase, gelatinase, (ACC) 1-aminocyclopropane-1-carboxylate deaminase, and indole-3-acetic acid (IAA), and also lead to nitrate reduction, phosphate solubilization, and assimilation of different carbon sources and siderophores (Compant et al. 2005; Kundan et al. 2015; Pathma and Sakthivel 2012; Przemieniecki et al. 2014, 2018).

In addition, our own research has shown that toxicogenic *Penicillium* spp. were suppressed by *Bacillus* spp., *Clostridium* spp., Actinomycetes metabolites, and fungi. Nevertheless, lower doses of vermicompost did not significantly increase the frequency of *Bacillus* spp. or some other bacterial groups, though an increase in *Bacillus* spp. able to produce antifungal metabolites was found. Our observations confirm the studies by Zhao et al. (2014), since we found a relationship between antibiotics and the number of *Bacillus* spp. as well as a decrease in phytopathogens, especially *Fusarium* spp., which remains another benefit of adding vermicompost.

Current research showed that the addition of vermicompost resulted in a reduction in carbon and a change in the C/N ratio to close to optimal values. Furthermore, C/N was inversely correlated with *Clostridium* spp., Actinomycetes and Pseudomonadaceae, and *Bacillus* spp., bacterial groups responsible for the decomposition of organic matter and nitrogen storage in soil. The above dependence resulted in an increase in biological life and biological processes in the soil which caused an increase in the concentration of soil enzymes and all nematode groups, with the exception of plant-parasitic nematodes, which did not increase along with the concentration of the added vermicompost. As demonstrated by Xiao et al. (2016) for three types of fertilizers (mineral, compost, and vermicompost), vermicompost was the most beneficial for reducing the population of *Meloidogyne incognita* in artificially infected tomatoes and reduced damage (by up to 77%). These results were consistent with the findings by Renčo and Kováčik (2015), Seenivasan and Poornima (2010), and Pandey (2005), where vermicomposts produced from different kinds of wastes significantly reduced the populations of plant-parasitic nematodes. We showed the same results under nursery conditions for the cultivation of larch. Vermicompost treatment could adhere to the IPM principle in limiting phytophagous nematodes in various crops. A clear relationship between the size of the nematode community and plant biomass has already been stated. Gebremikael et al. (2016) demonstrated, in conditions without vermicompost, on soil with natural microbiome and model plants that the presence of nematodes significantly increased plant biomass production (+ 9%), net nitrogen (+ 25%), and phosphorus (+ 23%) availability compared with their absence. This proves the important role of useful nematode groups in shaping flora and increasing nutrient availability. In this work, the observed synergy between nitrogen and phosphorus growth and the nematode community caused by the addition of vermicompost emphasizes the positive aspect of such fertilization in relation to a standard mineral one. This is further proof that vermicompost can play a key role for sustainable intensification.

Vermicompost in this test used in low doses also improved soil enzymatic activity. There are many studies indicating the improvement of the microbiological quality of soil with a simultaneous increase in enzymatic activity. Uz and Tavali (2014) have shown, in terms of enzymology, that the addition of vermicompost to alkaline soil already has a significant effect at a dose of 10 t ha^−1^, and the use of a four-time larger dose does not result in a very visible improvement in enzymatic activity or an increase in the number of bacteria. This indicates that even moderate doses of vermicompost, as in this test, can have a beneficial effect on enzymatic activity.

No correlation between changes in the ALP activity and other parameters was observed in this research. However, for ACP and IPP, the two enzymes associated with phosphorus transformation were the largest in the soil with the largest addition of vermicompost. Margalef et al. (2017), in phosphatase activity studies, found that one of the most important determinants of this class of enzymes was the availability of phosphorus-rich organic matter and total nitrogen content. In the current study, the activity of most enzymes was correlated with the total nitrogen content. We observed that nitrogen in the nitrate form may be a predictor of enzymatic activity, although it may be a synergistic effect due to the most favourable concentrations of potassium and phosphorus ions strongly correlated with this form of nitrogen.

Our own results demonstrated that the addition of vermicompost to the soil improved the chemical, biochemical, and biological properties of the substrates after 12 months of cultivation of *Larix* seedlings. An increase in the content of macroelements, as well as a more favourable microbiome and level of antibiotics; an increase in the number of beneficial nematodes; and an increase in soil enzymes involved in the transformation of phosphorus, carbon, and nitrogen are important soil properties affecting its health and productivity. At the same time, we are faced with a problem of global importance regarding the management of waste, from which vermicompost is produced. These are two reasons why the value of the technology of vermicomposting needs to be better appreciated and made more popular, even at the level of domestic households.

## Conclusions

In these studies, a beneficial cross-related effect was observed, associated primarily with the improvement of soil chemical parameters (including C/N value) which, in turn, improved the microbiological and nematological state of the soil along with the applied dose of vermicompost. Additives of vermicompost influenced bacteria, especially *Bacillus* spp. abundance (including Bacilli-produced antimicrobial compounds), along with a high level of other bacteria such as Actinomycetes and Pseudomonadaceae with a beneficial effect on soil. This situation decreased the level of undesirable fungi and plant-parasitic nematodes, while it increased the number of beneficial nematoda and the activity of soil enzymes. Considering utilization of waste, vermicomposting is a sustainable technology in the field of household waste management and for the natural strengthening of soil productivity in many areas, including forestry.

## Electronic supplementary material

ESM 1. Supplementary material. The presence (+) of genes responsible for the production of antibiotics with fungistatic activity. 1 = control soil (C), 2 = V10, 3 = V20, M = negative control (mastermix + sterile demineralized water) (PNG 409 kb)

High Resolution Image (TIFF 1877 kb)

## References

[CR1] Abdelmagid HM, Tabatabai MA (1987). Nitrate reductase activity of soils. Soil Biol Biochem.

[CR2] Aksakal EL, Sari S, Angin I (2016). Effects of vermicompost application on soil aggregation and certain physical properties. Land Degrad Dev.

[CR3] Andrássy I (2007) Free-living nematodes of Hungary (Nematoda errantia), II. In: Csuzdi C, Mahunka S (ed) Pedozoologica Hungarica No. 4. Hungarian Natural History Museum. Budapest, Hungary

[CR4] Arancon NQ, Galvis PA, Edwards CA (2005). Suppression of insect pest populations and damage to plants by vermicomposts. Bioresour Technol.

[CR5] Atik A (2014). Effect of different concentrations of vermicompost (Biohumus) on the root collar diameter and height growth in the seedlings of Anatolian Black Pine. J For.

[CR6] Atik A, Yılmaz B (2014). Effects of treatment with vermicompost on the some morphological and physiological characteristics of scots pine (*Pinus sylvestris* L.). Eur J Soil Sci.

[CR7] Atiyeh RM, Lee S, Edwards CA, Arancon NQ, Metzger JD (2002). The influence of humic acids derived from earthworm-processed organic wastes on plant growth. Bioresour Technol.

[CR8] Ayuso-Sacido A, Genilloud O (2005). New PCR primers for the screening of NRPS and PKS-I systems in Actinomycetes: detection and distribution of these biosynthetic gene sequences in major taxonomic groups. Microb Ecol.

[CR9] Bachman GR, Metzger JD (2007). Physical and chemical characteristics of a commercial potting substrate amended with vermicompost produced from two different manure sources. Horttechnology.

[CR10] Blouin M, Zuily-Fodil Y, Pham-Thi AT, Laffray D, Reversat G, Pando A, Tondoh J, Lavelle P (2005). Below-ground organism activities affect plant above-ground phenotype, inducing plant tolerance to parasites. Ecol Lett.

[CR11] Bouché M (1977). Strategies lombriciennes. Ecol Bull.

[CR12] Brzeski MW (1998). Nematodes of Tylenchida in Poland and temperate Europe. Museum and Institute of Zoology.

[CR13] Burns RG, DeForest JL, Marxsen J, Sinsabaugh RL, Stromberger ME, Wallenstein MD, Weintraub MN, Zoppini A (2013). Soil enzymes in a changing environment: current knowledge and future directions. Soil Biol Biochem.

[CR14] Cardoza YJ (2011). *Arabidopsis thaliana* resistance to insects, mediated by an earthworm-produced organic soil amendment. Pest Manag Sci.

[CR15] Casida L, Klein D, Santoro T (1964). Soil dehydrogenase activity. Soil Sci.

[CR16] Compant S, Duffy B, Nowak J, Clément C, Barka EA (2005). Use of plant growth-promoting bacteria for biocontrol of plant diseases: principles, mechanisms of action, and future prospects. Appl Environ Microbiol.

[CR17] Dick WA, Tabatabai MA (1978). Inorganic pyrophosphatase activity of soils. Soil Biol Biochem.

[CR18] Edwards CA (2004). Earthworm ecology.

[CR19] Eivazi F, Tabatabai MA (1988). Glucosidases and galactosidases in soils. Soil Biol Biochem.

[CR20] FAO and ITPS (2015) Status of the World’s Soil Resources (SWSR)–main report. Food and Agriculture Organization of the United Nations and Intergovernmental Technical Panel on Soils, Rome, Italy

[CR21] Flegel M, Schrader S (2000). Importance of food selected enzyme activities in earthworm casts (*Dendrabaema octaedra*, Lumbricidae). Soil Biol Biochem.

[CR22] Gebremikael MT, Steel H, Buchan D, Bert W, De Neve S (2016). Nematodes enhance plant growth and nutrient uptake under C and N-rich conditions. Sci Rep.

[CR23] Hu J, Wei Z, Friman V-P, Gu S-H, Wang X-F, Eisenhauer N, Yang T-J, Ma J, Shen Q-R, Xu Y-C, Jousset A (2016). Probiotic diversity enhances rhizosphere microbiome function and plant disease suppression. mBio.

[CR24] Hussain N, Abbasi T, Abbasi SA (2017). Detoxification of parthenium (*Parthenium hysterophorus*) and its metamorphosis into an organic fertilizer and biopesticide. Bioresour Bioprocess.

[CR25] Jacoby R, Peukert M, Succurro A, Koprivova A, Kopriva S (2017). The role of soil microorganisms in plant mineral nutrition-current knowledge and future directions. Front Plant Sci.

[CR26] Kabała C, Karczewska A (2019). Methodology for laboratory analysis of soils and plants (in Polish).

[CR27] Kabała C, Charzyński P, Chodorowski J, Drewnik M, Glina B, Greinert A, Hulisz P, Jankowski M, Jonczak J, Łabaz B, Łachacz A, Marzec M, Mendyk Ł, Musiał P, Musielok Ł, Smreczak B, Sowiński P, Świtoniak M, Uzarowicz Ł, Waroszewski J (2019). Polish soil classification, 6th edition–principles, classification scheme and correlations. Soil Sci Annu.

[CR28] Kandeler E, Gerber H (1988). Short-term assay of soil urease activity using colorimetric determination of ammonium. Biol Fertil Soils.

[CR29] Kolbe AR, Aira M, Gómez-Brandón M, Pérez-Losada M, Domínguez J (2019). Bacterial succession and functional diversity during vermicomposting of the white grape marc *Vitis vinifera* v. Albariño. Sci Rep.

[CR30] Kundan R, Pant G, Jadon N, Agrawal PK (2015). Plant growth promoting rhizobacteria: mechanism and current prospective. J Fertil Pestic.

[CR31] Ladd JN, Butler JHA (1972). Short-term assays of soil proteolytic enzyme activities using proteins and dipeptide derivatives as substrates. Soil Biol Biochem.

[CR32] Lazcano C, Sampedro L, Zas R, Domínguez J (2010). Vermicompost enhances germination of the maritime pine (*Pinus pinaster* Ait.). New For.

[CR33] Lehman RM, Cambardella CA, Stott DE, Acosta-Martinez V, Manter DK, Buyer JS, Maul JE, Smith JL, Collins HP, Halvorson JJ, Kremer RJ, Lundgren JG, Ducey TF, Jin VL, Karlen DL (2015). Understanding and enhancing soil biological health: the solution for reversing soil degradation. Sustainability.

[CR34] Lim SL, Wu TY, Lim PN, Shak KPY (2015). The use of vermicompost in organic farming: overview, effects on soil and economics. J Sci Food Agric.

[CR35] Lim S, Lee LH, Wu TY (2016). Sustainability of using composting and vermicomposting technologies for organic solid waste biotransformation: recent overview, greenhouse gases emissions and economic analysis. J Clean Prod.

[CR36] Margalef O, Sardans J, Fernández-Martínez M, Molowny-Horas R, Janssens IA, Ciais P, Goll D, Richter A, Obersteiner M, Asensio D, Peñuelas J (2017). Global patterns of phosphatase activity in natural soils. Sci Rep.

[CR37] Martin KJ, Rygiewicz PT (2005). Fungal-specific PCR primers developed for analysis of the ITS region of environmental DNA extracts. BMC Microbiol.

[CR38] McSpadden Gardener BB, Mavrodi DV, ThomashowLS WDM (2001). A rapid polymerase chain reaction-based assay characterizing rhizosphere populations of 2,4-diacetylphloroglucinol-producing bacteria. Phytopathology.

[CR39] Mora I, Cabrefiga J, Montesinos E (2011). Antimicrobial peptide genes in *Bacillus* strains from plant environments. Int Microbiol.

[CR40] Neher DA, Weicht TR, Bates ST, Leff JW, Fierer N (2013). Changes in bacterial and fungal communities across compost recipes, preparation methods, and composting times. PLoS One.

[CR41] Pandey R (2005). Management of *Meloigodyne incognita* in *Artemisia pallens* with bioorganics. Phytoparasitica.

[CR42] Pathma J, Sakthivel N (2012). Microbial diversity of vermicompost bacteria that exhibit useful agricultural traits and waste management potential. Springerplus.

[CR43] Pereira Mde G, Neta LC, Fontes MP, Souza AN, Matos TC, Sachdev Rde L, dos Santos AV, da Guarda Souza MO, de Andrade MV, Paulo GM, Ribeiro JN, Ribeiro AV (2014). An overview of the environmental applicability of vermicompost: from wastewater treatment to the development of sensitive analytical methods. Sci World J.

[CR44] Pérez-Piqueres A, Moreno R, López-Martínez M, Albiach R, Ribó M, Canet-Castelló R (2018). Composts and organic by-products in *Pinus halepensis* forestry. Front Sustain Food Syst.

[CR45] Perucci P, Casucci C, Dumontet S (2000). An improved method to evaluate o-diphenol oxidase activity of soil. Soil Biol Biochem.

[CR46] Pirsaheb M, Khosravi T, Sharafi K (2013). Domestic scale vermicomposting for solid waste management. Int J Recycl Org Waste Agricult.

[CR47] Przemieniecki SW, Kurowski TP, Korzekwa K, Karwowska A (2014). The effect of psychrotrophic bacteria isolated from the root zone of winter wheat on selected biotic and abiotic factors. J Plant Protect Res.

[CR48] Przemieniecki SW, Kurowski TP, Damszel M, Krawczyk K, Karwowska A (2018). Effectiveness of the *Bacillus* sp. SP-A9 strain as a biological control agent for spring wheat (*Triticum aestivum* L.). JAST.

[CR49] Rajiv P, Narendhran S, Kumar MS, Sankar A, Rajeshwari S, Rajendran V (2013). *Parthenium hysterophorus* L. compost: assessment of its physical properties and allelopathic effect on germination and growth of *Arachis hypogeae* L. Int Res J Environ Sci.

[CR50] Renčo M, Kováčik P (2015). Assessment of the nematicidal potential of vermicompost, vermicompost tea, and urea application on the potato-cyst nematodes *Globodera rostochiensis* and *Globodera pallida*. J Plant Protect Res.

[CR51] Schimel J, Becerra CA, Blankinship J (2017). Estimating decay dynamics for enzyme activities in soils from different ecosystems. Soil Biol Biochem.

[CR52] Schnerr H, Niessen L, Vogel RF (2001). Real time detection of the tri5 gene in Fusarium species by lightcycler-PCR using SYBR Green I for continuous fluorescence monitoring. Int J Food Microbiol.

[CR53] Seenivasan N, Poornima K (2010). Bio-management of root-knot nematode, *Meloidogyne incognita* (Kofoid and White) Chitwood in jasmine (*Jasminum sambac* L.). Pest Manag Horticult Ecosyst.

[CR54] Seinhorst JW (1962). On the killing, fixation and transferring to glycerin of nematodes. Nematologica.

[CR55] Silpa K, Yao L, Bhada-Tata P, Van Woerden F (2018) What a waste 2.0: a global snapshot of solid waste management to 2050. Urban Development Series. Washington, DC: World Bank. 10.1596/978-1-4648-1329-0

[CR56] Sinha RK, Valani D, Soni BK, Chandran V (2011). Earthworm vermicompost. A sustainable alternative to chemical fertilizers for organic farming.

[CR57] Song Y, Liu C, Finegold SM (2004). Real-time PCR quantitation of clostridia in feces of autistic children. Appl Environ Microbiol.

[CR58] Suanthie Y, Cousin MA, Woloshuk CP (2009). Multiplex real-time PCR for detection and quantification of mycotoxigenic *Aspergillus*, *Penicillium* and *Fusarium*. J Stored Prod Res.

[CR59] Svercel M, Duffy B, Dèfago G (2007). PCR amplification of hydrogen cyanide biosynthetic locus hcnAB in *Pseudomonas* spp. J Microbiol Methods.

[CR60] Szczech M, Rondomański W, Brzeski MW, Smolińska U, Kotowski JF (1993). Suppressive effect of a commercial earthworm compost on some root infecting pathogens of cabbage and tomato. Biol Agric Hortic.

[CR61] Tabatabai MA, Bremner JM (1969). Use of p-nitrophenyl phosphate for assay of soil phosphatase activity. Soil Biol Biochem.

[CR62] Uz I, Tavali IE (2014). Short-term effect of vermicompost application on biological properties of an alkaline soil with high lime content from Mediterranean region of Turkey. Sci World J.

[CR63] Venkatesh RM, Eevera T (2008). Mass reduction and recovery of nutrients through vermicomposting of fly ash. Appl Ecol Environ Res.

[CR64] Weber J, Karczewska A, Drozd J, Licznar M, Licznar S, Jamroz E, Kocowicz A (2007). Agricultural and ecological aspects of a sandy soil as affected by the application of municipal solid waste composts. Soil Biol Biochem.

[CR65] Xiao Z, Liu M, Jiang L, Chen X, Griffiths BS, Li H, Hu F (2016). Vermicompost increases defense against root-knot nematode (*Meloidogyne incognita*) in tomato plants. Appl Soil Ecol.

[CR66] Yasir M, Aslam Z, Kim SW, Lee SW, Jeon CO, Chung YR (2009). Bacterial community composition and chitinase gene diversity of vermicompost with antifungal activity. Bioresour Technol.

[CR67] Yu Y, Lee C, Kim J, Hwang S (2005). Group-specific primer and probe sets to detect methanogenic communities using quantitative real-time polymerase chain reaction. Biotechnol Bioeng.

[CR68] Zapałowska A, Skwiercz AT (2018). Populations of parasitic nematodes colonizing Jerusalem artichoke (*Helianthus tuberosus* L.). Acta Soc Bot Pol.

[CR69] Zhao Y, Selvaraj J, Xing F, Zhou L, Wang Y, Song H, Tan X, Sun L, Sangare L, Folly Y, Liu Y (2014). Antagonistic action of *Bacillus subtilis* strain SG6 on *Fusarium graminearum*. PLoS One.

