# Supplementary figures and images for: An evaluation of selected chemical, biochemical, and biological parameters of soil enriched with vermicompost
Source: Environ Sci Pollut Res Int. 2020 Oct 13;28(7):8117–27. doi: 10.1007/s11356-020-10981-z (PMC7854409; doi:10.1007/s11356-020-10981-z)

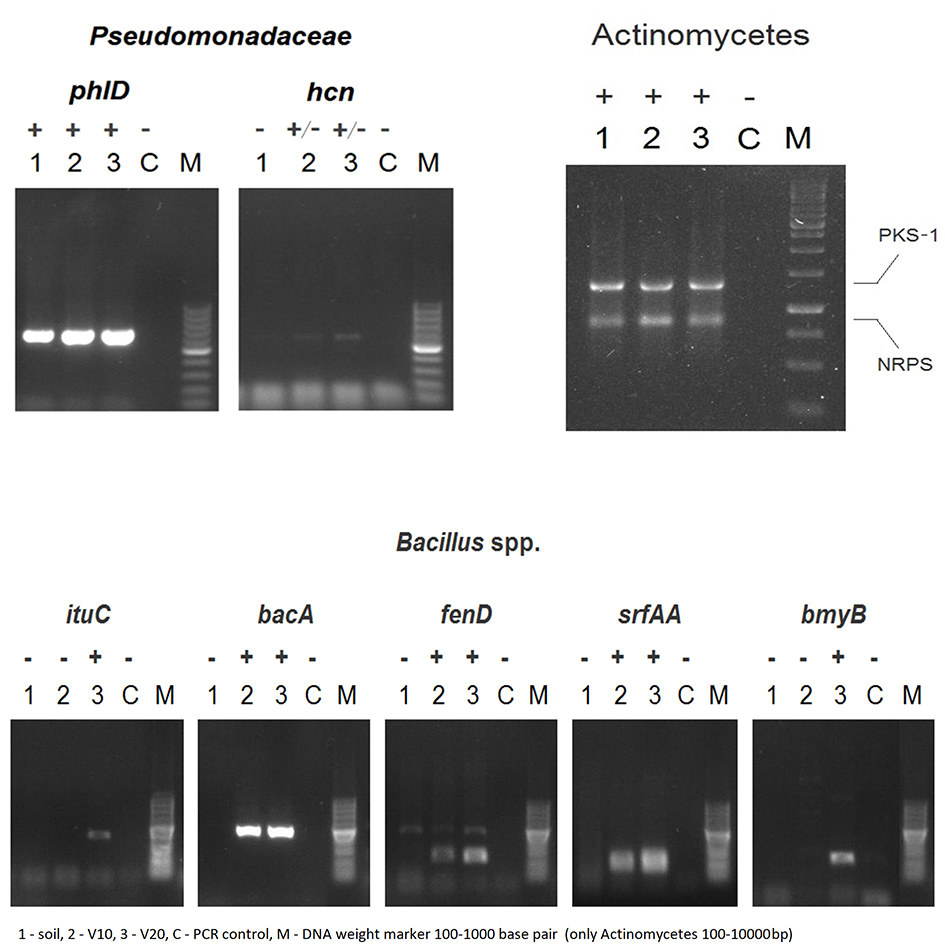

Supplement: Supplementary file 1 — Supplementary material. The presence (+) of genes responsible for the production of antibiotics with fungistatic activity. 1 = control soil (C), 2 = V10, 3 = V20, M = negative control (mastermix + sterile demineralized water) (PNG 409 kb) [file 11356_2020_10981_Fig2_ESM.png]

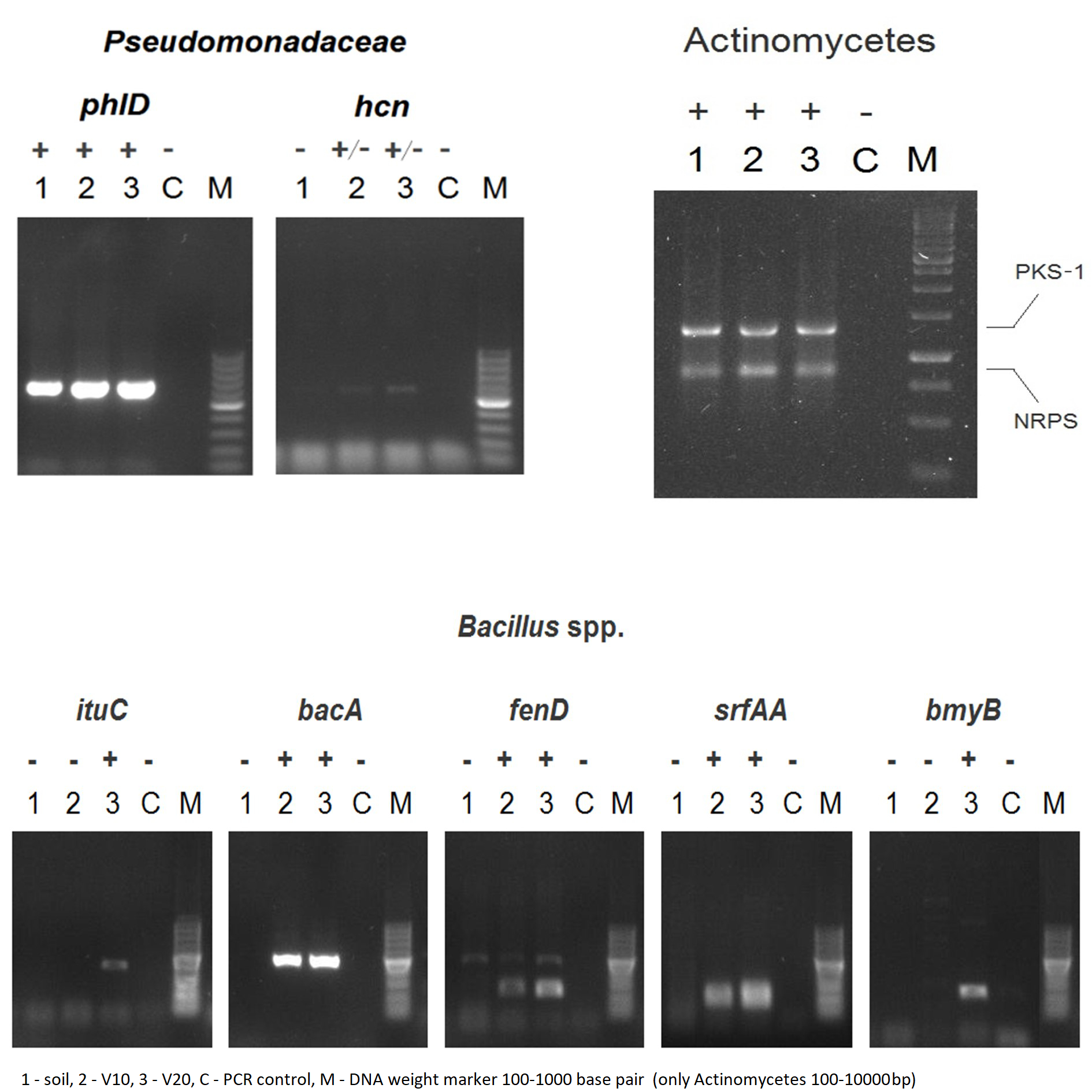

Supplement: Supplementary file 2 — High Resolution Image (TIFF 1877 kb) [file 11356_2020_10981_MOESM1_ESM.tiff]
